# Validity and reliability of the Persian version of illness invalidation inventory (3*I) among patients with non-inflammatory rheumatic painful disorders

**DOI:** 10.1186/s41927-022-00256-0

**Published:** 2022-05-03

**Authors:** Banafsheh Ghavidel-Parsa, Mohammad-Javad Khosousi, Sepehr Tohidi, Ali Bidari, Saman Soltani, Habib Zayeni, Ali Montazeri

**Affiliations:** 1 Rheumatology Research Center, Razi Hospital, School of Medicine, Guilan University of Medical Sciences, Rasht, Iran; 2 Gastrointestinal and Liver Diseases Research Center, Guilan University of Medical Sciences, Rasht, Iran; 3 Student Research Committee, School of Medicine, Guilan University of Medical Sciences, Rasht, Iran; 4 Department of Rheumatology, Iran University of Medical Sciences, Tehran, Iran; 5 Health Metrics Research Centre, Institute for Health Sciences Research, ACECR, Tehran, Iran; 6 Faculty of Humanity Sciences, University of Science and Culture, Tehran, Iran

**Keywords:** Pain, Fibromyalgia, Invalidation, Chronic musculoskeletal pain, Validation

## Abstract

**Background:**

The Invalidation Illness Inventory (3*I) is an instrument that assesses invalidation (including discounting and lack of understanding dimensions) experienced by patients with rheumatic disorders. This study aimed to translate and validate the 3*I in Iran.

**Methods:**

Following translation of the 3*I into the Iranian language (Persian), a cross-sectional study was conducted. A consecutive sample of females with chronic non-inflammatory rheumatic painful diseases completed the questionnaire. Patients also completed the Revised Symptom Impact Questionnaire (SIQR) and the Short Form Health Survey-12 (SF-12). To examine convergent validity, the correlation between the 3*I, the SIQR, and the SF-12 was assessed. The reliability of the 3*I was examined by internal consistency (the Cronbach's alpha coefficient) and intraclass correlation coefficient (ICC).

**Results:**

In all 196 patients participated in the study. The mean (SD) age of patients was 45.62 ± 10.70 years. Several significant correlations between the Invalidation Illness Inventory (discounting/lack of understanding) with the symptom impact (SIQR) and the short form health survey (SF-12) were observed lending support to the convergent validity of the 3*I. The Cronbach’s alpha coefficients were acceptable for most dimensions and sources, ranging from 0.52 to 0.88. Most ICC values for the dimensions of 3*I were above 0.75.

**Conclusions:**

The findings indicated that the Persian version of Illness Invalidation Inventory (3*I) is a valid instrument for invalidation assessment in patients with chronic pain. Given the high frequency of perceived invalidation among patients with rheumatic painful disorders, serious attention is needed to the issue in clinical and research settings.

## Background

Living with rheumatic diseases and chronic pain disorders are very debilitating and frustrating conditions, owing to the presence of inherent invisible symptoms such as pain, fatigue, and stiffness in these conditions. Subjective nature of rheumatic symptoms besides the absence of convincing explain in radiological and laboratory investigations, may lead to disbelief and distrust about the legitimacy of patients’ illness and consequently, misunderstanding and rejection of patients. This condition has recently been described as the ‘invalidation’ [1] which has been highly reported in patients with chronic pain and fibromyalgia (FM). The invalidation seems to reach to its maximum in the FM because of pure subjective nature of the presenting symptoms such as chronic widespread pain, fatigue, unrefreshed sleep, and cognitive symptoms in absence of any pathological or laboratory findings that could explain the symptoms [2–4].

The illness invalidation has been suggested to have a damaging effect on psychological health [5] and contributes to intensify pain [6, 7] and disability. The perceived invalidation by patients can lead to negative social responses, particularly discounting (rejecting) and also lack of positive social response (not being acknowledged) [1, 3]. These negative social interactions could influence mental and physical health [8], clinical disease severity, and disability [4, 7]. Moreover, the effect of invalidation on pain and health status seems to be mutual. Expectedly, the worsening of illness with increased pain and disability creates a vicious cycle that aggravates invalidation perception and vice versa.

The influential impact of invalidation on clinical severity, quality of life, health behavior, therapy adherence, and outcome makes it as a prominent theme in the care of fibromyalgia and chronic pain disorders [3, 4, 8–10]. Even so, the invalidation is a neglected problematic issue in care of majority of chronic pain disorders especially FM [1, 11]. While the invalidation concept has been taken into account in rheumatic diseases, it remains to be ignored in clinical practice and researches. It might be partly related to the absence of a valid and reliable instrument for measurement of invalidation. Invalidation Illness Inventory (3*I) has proposed a novel tool to measure invalidation dimensions in rheumatic patients [1]. The 3*I have been assessed across different languages including Dutch, English, French, German, Portuguese, Spanish [12] and Swedish [13]. The initial version of the questioner was developed in the Netherlands and then the English version was provided. Then, the English version of the questionnaire was used for the translation to other languages named above [12]. However, it is not validated in Iran yet. Thus this study aimed to translate and validate the questionnaire in Iran.

## Methods

### Invalidation illness inventory (3*I)

**Fig. 1:**
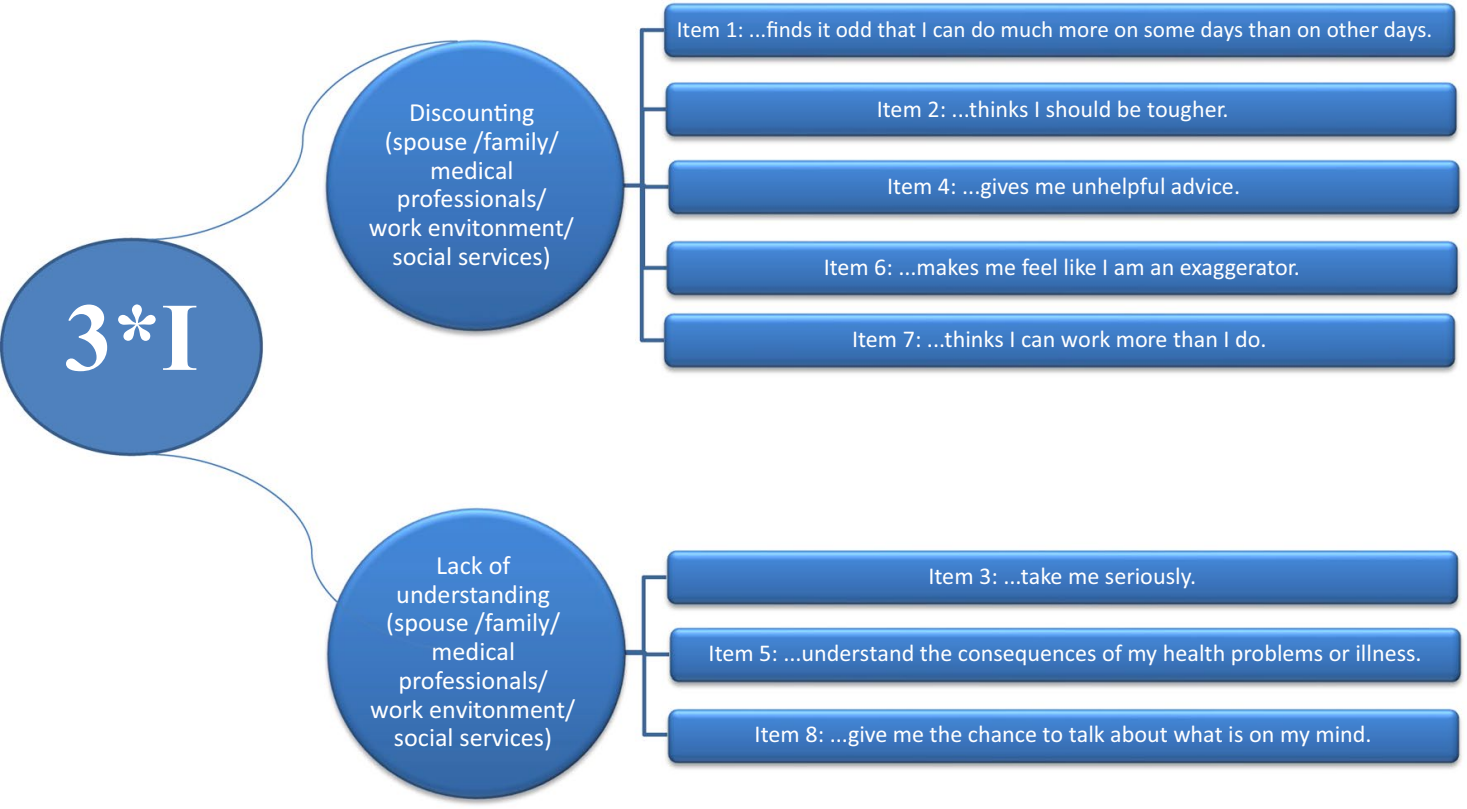
A schematic diagram of the Invalidation Illness Inventory (3*I)

It consists of two dimensions: discounting and lack of understanding (Fig. 1) that could be derived from five different sources, including spouses, family, colleagues, health professionals, and the community [1, 3]. In fact, the questionnaire has 40-items (8 items for each 5 sources). The discounting dimension of 3*I evaluates active negative social responses (denying, lecturing, and overprotecting) that reflects social rejection of patients in their personal relationships. The lack of understanding dimension, represents lack of positive social responses (supporting and acknowledging) that reflects lack of social support. Each item is rated on a 5-point scale (1 = never, 2 = seldom, 3 = sometimes, 4 = often, 5 = very often) [3].

### Translation

Following obtaining permission from Dr. Marianne B. Kool, the 3*I was translated into Persian using forward–backward procedure. The questionnaire was translated independently into Persian by two bilingual general physicians and then a consolidated Persian was provided. Next, it was back translated into English by two bilingual rheumatologist (BGH) and internal medicine specialist. Finally, the back translation of 3*I was compared with the original instrument in order to confirm equivalence between both versions in which no differences were found. At this stage to ensure content and face validity an expert pant evaluated the questionnaire and found it satisfactory. In addition, four patients completed the questionnaire and reported that it reads well. They indicated that they could understand the wordings and could respond to the questionnaire easily. As such provisional version of the questionnaire was provided and subjected to psychometric evaluation.

### The psychometric evaluation

A cross sectional study was conducted on a sample of patients with chronic non-inflammatory musculoskeletal pain who were seen in the rheumatology clinic of Razi hospital affiliated to Guilan University of Medical Sciences, Rasht, Iran from March 2018 to July 2019. All participants were female and were enrolled consequently by a rheumatologist (BGH) experienced in chronic pain and fibromyalgia diagnosis and management. Almost all patients attending this clinic are females. The diagnosis was based on the 2016 American College of Rheumatology (ACR) criteria [14, 15]. The patients with non-inflammatory rheumatic conditions such as FM, osteoarthritis, non-specific low back pain, mechanical neck pain, myofascial pain or tendinitis (such as lateral or medial epicondylitis, adhesive capsulitis, etc.) were included. Patients were excluded if they had an inflammatory rheumatic disease, severe depression, or history of antidepressant drug consumption for at least 6 weeks, having known confounding medical illness (such as malignancy, disabling medical condition) at the time of enrollment and inability to read or write. Some terms and concepts used in the questionnaire such as "discounting" and "lack of understanding" were completely explained to the patients.

### Additional measures

In addition to collecting information on demographic data such as age, marital status, educational level, work status and disease duration, the following questionnaire also were administered.

1. The Symptom Impact Questionnaire-Revised (SIQR): The validated Persian version of the SIQR [16] was used as an instrument assessing disease impact on daily life and clinical severity in FM and other chronic pain rheumatic diseases [17]. It contains 21 questions. All questions are based on an 11-point numeric rating scale ranging from 0 (none) to 10 denoting the worst. The questionnaire includes three sets of domains: function, overall impact and symptoms. The total SIQR scores would then be the sum of the three domain scores. The higher score indicates higher symptom impact.
2. The Short Form Health Survey (SF-12): Health status and quality of life was assessed by the validated Persian version of SF-12. The questionnaire includes eight subscales: physical functioning, role physical, social functioning, role emotional, bodily pain, general health, vitality, and mental health. Scores on each subscale range from 0 to 100 with lower scores indicating worse possible conditions [18].

### Statistical analysis

#### Validity

Validity was assessed by convergent validity. To evaluate convergent validity, the correlation between the 3*I and the SIQR and the SF-12 were assessed using the Spearman's correlation coefficient. Correlation values of 0.40 or above were considered satisfactory (r = 0.81–1.0 as excellent, 0.61–0.80 very good, 0.41–0.60 good, 0.21–0.40 fair, and 0–0.20 poor). [19].

#### Reliability

Reliability was assessed by internal consistency and reproducibility. The Cronbach's alpha coefficient was used to determine internal consistency. Values above 0.7 was considered as satisfactory internal consistency for the questionnaire.

#### Stability

Test–retest analysis was performed to evaluate the intraclass correlation coefficient (ICC). The values between 0.70 and 0.9, and greater than 0.9 were considered as good, and excellent stability, respectively [20].

## Results

### Patients

In all 196 patients were entered into the study. The mean age of participants was 45.62 ± 10.70 years. The baseline characteristics of the patients are summarized in Table 1.

**Table 1:** Demographic and baseline characteristics of patients

	**Statistics**
**Age (year)**	
Mean ± SD	45.6 ± 10.70
Range	18–73
**Marital status**	
Single	36 (18.4%)
Married	160 (81.6%)
**Education**	
Primary	53 (27%)
Secondary	86 (43.9%)
Higher	57 (29.1%)
**Employment**	
Employed	34 (17.3%)
Unemployed	162 (82.7%)
**Diagnosis**	
Fibromyalgia	134 (68.4%)
Periarthritis	13 (6.6%)
Osteoarthritis	30 (15.3%)
Low Back Pain	7 (3.6%)
Others	12 (6.1%)
**Time since diagnosis (month)**	
Fibromyalgia (means ± SD)	46.37 ± 61.83
Other diseases (means ± SD)	16.61 ± 20.64

### Invalidation experiences

The frequency of patients with chronic pain who sometimes (> 2.5–3.5) and often/very often (> 3.5–5) experienced discounting by their spouse, family, medical professionals, work environment, and social services were 20.95%, 28.81%, 32.73%, 23.53%, and 11.11%, respectively. Likewise, the frequency of patients with chronic pain who sometimes (> 2.5–3.5) and often/very often (> 3.5–5) experienced lack of understanding by their spouse, family, medical professionals, work environment and social services were 26.75%, 12.22%, 26.74%, 21.05%, and 5.55%, respectively.

### Validity

Significant correlations between the discounting and lack of understanding and the disease impact (SIQR) and the health survey (SF-12) were observed. Overall, discounting by spouse and family correlated most strongly with disease impact and health survey. The correlations of discounting with total SIQR score and mental health score were higher than other subscales. Discounting and lack of understanding by medical professionals hardly correlated with the SF-12 scores. The results are shown in Tables 2 and 3.

**Table 2:** Spearman's correlations between the Persian 3*I (discounting and lack of understanding) and the SIQR

	SIQR domains			
	Functional	Overall impact	Symptom	Total
**Spouse**				
Discounting	0.35**	0.40**	0.46**	0.47**
Lack of understanding	0.30**	0.26*	0.30	0.32**
**Family**				
Discounting	0.38**	0.35**	0.43**	0.44**
Lack of understanding	0.27**	0.30**	0.24**	0.29**
**Medical professionals**				
Discounting	0.03	0.11	0.14	0.07
Lack of understanding	0.29**	0.21*	0.20	0.27*
**Work environment**				
Discounting	0.52*	0.36	0.11	0.33
Lack of understanding	0.53*	0.50*	0.30	0.47*
**Social services**				
Discounting	0.32	0.15	0.02	0.16
Lack of understanding	0.51*	0.16	0.24	0.36

Positive correlation indicates that more discounting or lack of understanding is associated with worse functional, overall impact, and symptom scores

*p < 0.05 correlation significant at 0.05 level (two tailed)

**p < 0.01 correlation significant at 0.01 level (two tailed)

**Table 3:** Spearman's correlations between the Persian 3*I (discounting and lack of understanding) and the SF-12

	SF-12 subscales							
	PF	RP	BP	GH	VT	SF	RE	MH
**Spouse**								
Discounting	−0.39**	−0.25**	−0.30**	−0.23**	−0.23**	−0.22**	−0.37**	−0.40**
Lack of understanding	−0.30**	−0.23**	−0.25**	−0.18*	−0.21**	−0.28**	−0.33**	−0.30**
**Family**								
Discounting	−0.43**	−0.40**	−0.40**	−0.20**	−0.24**	−0.30**	−0.37**	−0.33**
Lack of understanding	−0.24**	−0.30**	−0.19**	−0.18*	−0.12	−0.31**	−0.26**	−0.11
**Medical professionals**								
Discounting	−0.15	−0.20	−0.19	−0.20	0.02	−0.15	−0.12	−0.05
Lack of understanding	−0.05	−0.02	−0.10	−0.18	−0.10	−0.00	−0.07	−0.04
**Work environment**								
Discounting	−0.53*	−0.67**	−0.56*	−0.14	0.00	−0.67**	−0.57*	−0.18
Lack of understanding	−0.62**	−0.72**	−0.42	−0.39	−0.29	−0.46*	−0.53*	−0.56*
**Social services**								
Discounting	−0.30	−0.48*	−0.15	0.25	0.17	−0.52*	−0.34	−0.12
Lack of understanding	−0.46	−0.24	−0.30	−0.06	0.14	−0.29	−0.50*	−0.40

*PF* physical functioning, *RP* role physical, *BP* bodily pain, *GH* general health, *VT* vitality, *SF* social functioning, *RE* role emotional, *MH* mental health

A negative correlation indicates that more discounting or lack of understanding is associated with worse mental health, worse physical health or social functioning

*p < 0.05 correlation significant at 0.05 level (two tailed)

**p  < 0.01 correlation significant at 0.01 level (two tailed)

### Reliability

The intraclass correlation coefficient (ICC) for the ‘discounting’ scores for the spouse, family, medical professionals, work environment, and social services on the first and second visits were 0.88, 0.87, 0.92, 0.74 and 0.91, respectively. The ICC for the ‘lack of understanding’ scores on two visits for the spouse, family, medical professionals, work environment, and social services were 0.82, 0.87, 0.70, 0.86, and 0.87, respectively. The Cronbach’s α coefficients of spouse discounting and family lack of understanding which were questionable, 0.57 and 0.52 respectively. The Cronbach’s α for the rest of ‘discounting’ and ‘lack of understanding’ items ranged from 0.60 to 0.88 indicating moderate to high values. All Cronbach's α and ICC values for 3*I are shown in Table 4.

**Table 4:** Mean values and standard deviation for each Persian 3*I item score, test–retest reliability by ICC and Cronbach's α for the questionnaire domains

Items	Visit one (Mean ± SD)	Visit two (Mean ± SD)	ICC	95% CI of ICC	Cronbach's alpha
Item 1	2.50 ± 1.28	2.29 ± 1.19	0.68*	(0.27, 0.86)	
Item 2	3.70 ± 1.26	4.04 ± 1.04	0.74*	(0.40, 0.88)	
Item 3	3.79 ± 1.10	4.12 ± 1.36	0.73*	(0.39, 0.88)	
Item 4	2.41 ± 1.21	2.16 ± 1.46	0.79*	(0.52, 0.91)	
Item 5	3.25 ± 1.29	3.20 ± 1.64	0.80*	(0.54, 0.91)	
Item 6	2.75 ± 1.42	2.54 ± 1.86	0.89*	(0.75, 0.95)	
Item 7	2.54 ± 1.28	2.16 ± 1.23	0.86*	(0.68, 0.94)	
Item 8	3.45 ± 1.25	3.70 ± 1.30	0.87*	(0.71, 0.94)	
*Discounting*	2.78 ± 0.80	2.64 ± 0.98	0.88*	(0.74, 0.95)	0.57
*Lack of understanding*	2.50 ± 0.90	2.31 ± 1.23	0.82*	(0.60, 0.92)	0.76
**Family**					
Item 1	2.92 ± 1.35	3.19 ± 1.38	0.82*	(0.61, 0.92)	
Item 2	3.28 ± 1.41	3.96 ± 1.37	0.81*	(0.60, 0.91)	
Item 3	3.67 ± 1.12	3.96 ± 1.37	0.80*	(0.58, 0.91)	
Item 4	2.21 ± 1.16	1.75 ± 1.20	0.74*	(0.44, 0.88)	
Item 5	3.39 ± 1.34	3.17 ± 1.44	0.77*	(0.51, 0.89)	
Item 6	2.85 ± 1.29	2.53 ± 1.62	0.88*	(0.75, 0.94)	
Item 7	2.92 ± 1.21	2.89 ± 1.49	0.83*	(0.63, 0.92)	
Item 8	3.53 ± 1.10	3.78 ± 1.03	0.79*	(0.55, 0.90)	
*Discounting*	2.84 ± 0.66	2.94 ± 0.86	0.87*	(0.73, 0.94)	0.65
*Lack of understanding*	2.46 ± 0.89	2.35 ± 1.14	0.87*	(0.73, 0.94)	0.52
**Medical professionals**					
Item 1	2.21 ± 1.37	1.67 ± 1.18	0.88*	(0.74, 0.94)	
Item 2	3.35 ± 1.33	3.53 ± 1.40	0.82*	(0.62, 0.91)	
Item 3	4.57 ± 0.83	4.71 ± 0.65	0.81*	(0.60, 0.91)	
Item 4	1.32 ± 0.66	1.42 ± 0.74	0.85*	(0.69, 0.93)	
Item 5	4.32 ± 0.94	4.60 ± 0.91	0.76*	(0.49, 0.89)	
Item 6	1.71 ± 1.08	1.82 ± 1.12	0.74*	(0.45, 0.88)	
Item 7	1.89 ± 0.91	2.10 ± 0.91	0.80*	(0.58, 0.91)	
Item 8	4.10 ± 1.13	4.67 ± 0.81	0.75*	(0.46, 0.88)	
*Discounting*	2.10 ± 0.61	2.11 ± 0.62	0.92*	(0.84, 0.96)	0.66
*Lack of understanding*	1.66 ± 0.66	1.33 ± 0.49	0.70*	(0.36, 0.86)	0.76
**Work environment**					
Item 1	2.92 ± 1.07	2.71 ± 1.43	0.88*	(0.65, 0.96)	
Item 2	4.14 ± 0.86	4.28 ± 0.72	0.87*	(0.60, 0.95)	
Item 3	3.71 ± 0.99	4.00 ± 0.96	0.84*	(0.50, 0.94)	
Item 4	2.21 ± 0.97	1.71 ± 0.91	0.86*	(0.58, 0.95)	
Item 5	2.71 ± 1.20	2.07 ± 1.54	0.72*	(0.15, 0.91)	
Item 6	2.71 ± 1.32	2.78 ± 1.80	0.89*	(0.65, 0.96)	
Item 7	3.57 ± 1.08	3.57 ± 1.08	0.89*	(0.66, 0.96)	
Item 8	4.07 ± 0.99	4.28 ± 0.72	0.80*	(0.40, 0.93)	
*Discounting*	3.11 ± 0.33	3.01 ± 0.44	0.74*	(0.20, 0.91)	0.88
*Lack of understanding*	2.50 ± 0.70	2.54 ± 0.80	0.86*	(0.57, 0.95)	0.61
**Social services**					
Item 1	1.91 ± 0.66	1.50 ± 0.79	0.86*	(0.51, 0.96)	
Item 2	3.08 ± 1.16	3.00 ± 1.20	0.73*	(0.08, 0.92)	
Item 3	4.00 ± 0.73	3.83 ± 1.02	0.80*	(0.33, 0.94)	
Item 4	1.58 ± 0.79	1.66 ± 0.77	0.77*	(0.23, 0.93)	
Item 5	3.16 ± 1.40	2.83 ± 1.40	0.86*	(0.51, 0.96)	
Item 6	2.16 ± 1.11	2.00 ± 1.20	0.89*	(0.63, 0.97)	
Item 7	2.41 ± 1.50	3.00 ± 1.34	0.86*	(0.51, 0.96)	
Item 8	3.50 ± 0.90	4.00 ± 1.20	0.78*	(0.23, 0.93)	
*Discounting*	2.23 ± 0.76	2.23 ± 0.71	0.91*	(0.68, 0.97)	0.71
*Lack of understanding*	2.44 ± 0.72	2.44 ± 0.67	0.87*	(0.55, 0.96)	0.75

*Correlation is significant at the 0.05 level

## Discussion

The study findings revealed that the Persian version of 3*I was a reliable and valid instrument for assessing invalidation among the Iranian population with chronic pain disorders. The findings also showed the high frequency of invalidation perception among patients with chronic pain disorders. The perceived invalidation especially stemming from the spouse and family sources correlated with worst disease impact and health status.

There is no questionnaire for description and measurement of invalidation in Iran. The current study was the first investigation that attempted to address the topic. A satisfactory level of reproducibility in all 3*I items and dimensions in five sources indicated that it is a reliable measure for evaluation of invalidation experiences by the different sources. Most values were above 0.75 and acceptable. The study by Kool et al. who introduced the 3*I for the first time did not evaluate test–retest reliability [3].

Levels of internal consistency were good and acceptable in the most dimensions and sources of 3*I (ranging from 0.61 to 0.88) which were comparable with the Kool’s study [3]. However, the levels of consistency of the Persian 3*I was lower than the Kool study in two sources (spouse discounting and family lack of understanding). These lower levels of consistency may be related to the heterogeneous patient population in our study which included the patients with FM and non-inflammatory chronic pain disorders such as osteoarthritis, low back pain and regional pain diseases versus those with relative homogenous rheumatic diseases (e.g., chronic low back pain, rheumatoid arthritis, and FM) in the previous validation studies [3, 7, 21]. This heterogeneity could lead to the differences in the symptomology and associated invalidation experiences within our papulation. Additionally, the significance of these lower values was undetermined, so the wording and concept of questions in the spouse and family sources of Persian 3*I were identical to other sources.

The convergent validity of Persian 3*I dimensions were evaluated by correlation between the SIQR domains and the SF-12 subscales. We found several moderate but significant correlations of both invalidation dimensions (the discounting and lack of understanding) with worse disease impact, physical functioning, more bodily pain, and poor mental health in the spouse and family sources. The strongest correlations were between the spouse and family sources of discounting/lack of understanding and different domains of the disease impact and health status. It is conceivable that spouse and family play a main role in relationships’ intimacy and consequently happiness and well-being feeling of patients. So, the invalidation stemming from these sources may be associated with a greater correlation with disease severity and health status. Consistent with previous studies, our results demonstrated the perceived invalidation from spouse and family was the more compelling indicators of poor disease impact and health status [3, 4, 7].

Interestingly, there were strong correlations of the invalidation perception derived from work environment with the functional domains of SIQR and SF-12. These significant correlations could just be seen in different functional aspects of the SIQR and the PF, RP, SF, and RF subscales of SF-12, but not for other subscales of these questionnaires. The more invalidation experienced from the work environment, the worse physical and social status and functioning were found. Such observations strongly lend support to satisfactory convergent validity in the functional properties of the Persian 3*I.

Invalidation by medical professionals correlated hardly with disease impact and health status. The low correlations were found between the SIQR domains and the lack of understanding dimension. It seems that the perceived invalidation from medical professionals might be due to different reason. For instance, it might be due to inability of medical providers to understand illness impact, misdiagnosis, or inability to control patients’ symptoms, rather than simple empathy between patients and their caregivers [22]. Usually patients with FM experience a long journey in achieving a correct diagnosis and management [23]. As the majority of our participants suffered from FM, the delayed diagnosis and mismanagement occurring in the FM patients was conceivable. The mean long duration of symptoms to diagnosis (46.37 months) in the FM papulation could be indirectly an indicator of this type of invalidation by medical professionals. In other word, the invalidation arising from medical professionals seems to be different and probably must be measured using another scale [24]. Moreover, the lack of understanding reflects not recognizing, comprehending, and emotionally supporting the patient or illness. Its meaning and concept are different from the discounting which represents disbelieving, admonishing, dismissing inability to work, and offering unusable advice [3]. Thus, our findings seem reasonable that showed the presence of meaningful correlations between misunderstanding from medical professionals with disease impact. The lack of understanding and rejection of patients with FM by medical caregivers have been known to occur frequently in clinical settings [22].

Additionally, in congruence with the previous studies, the discounting in all sources correlated more closely to the disease impact and health status rather than lack of understanding [3, 7]. This finding re-emphasizes that the different impact of overt rejection (discounting) versus not being acknowledged (lack of understanding) and also implies the importance of distinguishing these two aspects of invalidation in research and clinical assessment. However, overall these results indicated satisfactory construct validity for the Persian 3*I. The more invalidation experience by the patients was associated with worse physical function and mental well-being.

Interestingly, in our study, the extent of invalidation arising from the different sources was remarkable. Twenty to thirty percent of the patients experienced the invalidation ‘sometimes or often/very often’ from other people. Although frequent sources of invalidation are the spouse and work environment, there was considerable invalidation perception from other sources. It was in agreement with previous studies where it was shown that the extent of invalidation could differ from different sources [3, 7]. Although it remains to be clarified whether intimacy, and type of relationships and cultural factors could have differential impact on the invalidation derived from different sources, the spouse and family sources of invalidation appears to be more important and indicate higher influential effects on the quality of life in patient with chronic pain conditions. According to our results, the most frequent sources of perceived invalidation were from the close relatives and work environment which showed the most meaningful associations with the worse disease impact and health status. This data emphasizes the need for educating family and people who are interacting with patients closely. Perhaps this could empower patients to cope better with invalidation.

This study had some limitations. Only female patients were recruited and therefore the findings of this study cannot be generalized. Furthermore, 82% of our patients were not employed, which were generally less educated and more unemployed when compared with the previous studies. Although education, literacy levels, and lower socioeconomic status seem to change the likelihood of invalidation due to misinformed conceptions about pain [21], it remains to fully explore to what extent and how these demographic variables influence the impact of invalidation on physical and psychological health outcomes. Moreover, a large number of patients in this study did not complete the last two source categories (work environment and social services). Therefore, caution should be exercised in interpreting the findings of these two sources.

## Conclusion

The findings indicated that the Persian version of Illness Invalidation Inventory (3*I) is a valid instrument for the assessment of invalidation in patients with chronic pain. Given the high frequency of perceived invalidation among patients with rheumatic painful disorders, serious attention is needed to the issue in clinical and research settings.

## Data Availability

The datasets used and/or analyzed during the current study available from the corresponding author on reasonable request.

## Abbreviations

3*I: Illness Invalidation Inventory
SRQR: Symptom Impact Questionnaire-Revised
SF-12: 12-Item Shor form Health Survey
FM: Fibromyalgia
ICC: Intraclass correlation coefficient
